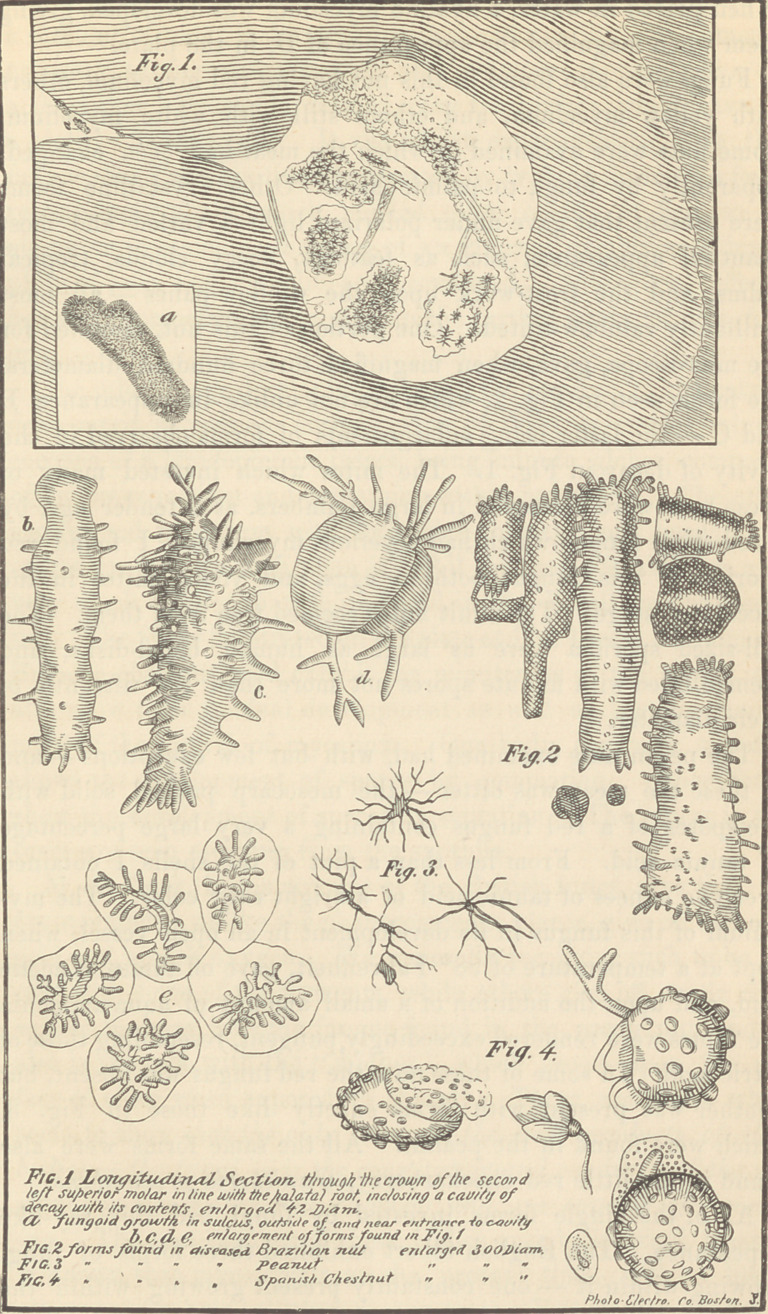# Report on Histology and Microscopy

**Published:** 1879-06

**Authors:** G. F. Waters


### Selections.

---

### ARTICLE XIII.

### REPORT ON HISTOLOGY AND MICROSCOPY. By DR. G. F. WATERS.

By reference to the Transactions of the American Dental Association for 1877, page 43, discussion of Dr. Dean's paper on Dental Physiology, it will be seen that I referred to some strange appearances found within a cavity (in a section) of a tooth. Fig. 1 of the accompanying plate gives a view of this portion of the section enlarged forty diameters, and having some portion of the contents idealized at eighty diameters. This cavity was in the enamel, in the grinding surface of the palatal portion of the crown of the first left superior molar, and opened from a sulcus into which small particles of food, during mastication, were forced; subsequent fermentation would displace some of the impacted mass only to be replaced again at the next meal. This series of operations had continued for forty years, when the tooth was extracted, so that the penetration of the enamel and formation of the cavity were very gradual.

As we look into this cavity we desire to know what and from whence are these curious bodies. In pursuing an investigation for the purpose of elucidating these questions, we naturally first look to the various kinds of food that have been ground upon the tooth, and in them we find forms that very nearly, if not exactly, correspond with those found in this cavity of decay; and we shall be surprised at the vast number and variety of the minute cryptogamous forms of life to be met with in all articles of food, particularly the fruits, nuts and roots. In the sweet potato I find forms developing before decay, resembling B (which is an enlargement of one of the forms found in the cavity in Fig. 1). They are dark but covered with spines more or less permeable by light, and having a length as to width of six or more to one. They appear before the mycelium of a well-known pencillium, and resemble in it no respect, and I trace no connection between the two, except that the spiny form seems always to precede the mycelium of this pencillium.

This mycelium is hyaline, smooth, of indefinite length, with many fine branches, and seldom exceeds the tenth of a millimeter in size. In growing, their course is between the cell-walls of the starch-grains, thus causing an old wilted potato to stiffen up and appear quite fresh. As they progress, sporidia-bearing filaments penetrate the starch-cells, changing their contents to carbonic acid, water and sporidia. Then, by the rupturing of the starch-cells, the potato suddenly becomes soft and is soon covered by white spore-bearing filaments, in which condition it presents the apperance of the body found in the sulcus in Fig. 1, marked A, enormously magnified, an enlarged view of which is given in the white square on the enamel. It will be seen that the potato may be considered good food, whilst largely composed of fungoid forms, mycelium and sporidia. When the potato is cooked — more especially when baked — the part infested by the fungi will be found harder and darker than the other parts, and also to give off a peculiarly disagreeable odor. Heat, sufficient to cook the potato, seems to destroy the vitality of the fungi, so that when thus eaten, they do not seem to disturb the digestion.

What I have said of these forms of fungi in the apparently healthy potato, M. C. Cook, in his "Rust, Smut, Mildew and Mould," has said of many other articles of food which I have been in the habit of eating (as the sweet potato) uncooked. Apples, pears, and peaches have — in addition to form alluded to above which grow within — other forms growing upon their surface, allied to lichens. In the beet, turnip and onion, whilst in an anticarious condition, fungoid forms were discovered resembling some of those found in this cavity, but which are not here illustrated. The castana or Brazil nut showed a large variety of fungi. In one of these, microscopic truffles, or bodies resembling the truffle, were found packed solidly throughout the entire mass.

FIG. 1 Longitudinal Section through the crown of the second left superior molar in line with the palatal root, inclosing a cavity of decay with its contents, enlarged 42 Diam.

a fungoid growth in sulcus, outside of and near entrance to cavity b, c, d, e, enlargement of forms found in Fig. 1

FIG. 2 forms found in diseased Brazilian nut enlarged 300 Diam.

FIG. 3 " " " " Peanut " " "

FIG. 4 " " " " Spanish Chestnut " " "

Photo-Electro. Co. Boston. J.

When placed in water, a hyaline mycelium was developed, giving them when magnified the appearance of D, in the plate.

Fungi were also found in this nut having red mycelium, others with yellow mycelium, and others still with white mycelium. Some nuts were examined in which the meat had been changed, apparently by fungi, to palmic acid. Other crystalline forms were present that gave under polarized light a varied and most beautiful appearance, such as feathers, fleecy clouds, tropical palms, and the frost-work upon the window-panes — all most brilliantly rainbow-tinted. One portion of this nut, mounted for the microscope, gave, when magnified three hundred diameters, the forms seen in Fig. 2, which are not unlike in appearance B and C — the latter being enlargements of forms observed in the cavity of decay in Fig. 1. The mites which invested many of these nuts were destroyed in large numbers, at a tender age, by a red spore fungus which had a yellow mycelium. I found red sporidia of this fungus both in eggs, and young mites in the sacculated stage. The adult mites seemed free from them. The full-sized sporidia were as large as human blood-disks, and seemed filled with minute spores not more than the fifteenth of their diameter.

The pecan nuts examined had, with but few exceptions — and in these the meat was bitter — the mesocarp packed solid with the spores of a red fungus containing a very large percentage of tannic acid. From less than a pint of the shells I obtained over two ounces of tannic acid of a bright ruby color. The mycelium of this fungus in its development in an open vessel, when kept at a temperature of 98° Fahrenheit, gave off fumes of acetic acid, and upon the addition of a amall amount of aqua-ammonia the fumes were rendered exceedingly pungent, resembling those of nitric acid. In some of these nuts the red fungus was absent, but another was present with forms exactly like those in Fig. 3, which were found in the peanut. All the same forms were also found in the little red checkerberry, *Gaultheria procumbens*.

All of the single forms invested by fungi had this ray-like appearance. The English filbert exhibited three forms of fungi growing within it — one constantly present growing within the substance of the cotyledons, the mycelium of which was so minute as not to be traceable in a microscopic section, but when sown in a tumbler of water it developed with great rapidity and formed a net-work throughout, giving it an opalescent appearance. In a short time a dense film formed upon the surface, which gave off a highly offensive odor. It was whilst experimenting with this fungus that I was six times prostrated by typhoid fever, or a fever resembling typhoid, which yielded quite readily at first to an aborative treatment, consisting of an internal exhibition of bisulphite of soda and carbolic acid combined. Subsequent effects, however, compelled me to give up the further study of this fungus.

The other fungi developing between the pericarp of the cotyledons merit a passing notice. One of them resembles minute cocoons of a lepidopterous insect, being minute globes composed of yellow mycelium and containing within red spores. The other showed red mycelium with long club-shaped spore heads with spores growing in sulci upon it. All the spores in this nut were very much smaller than those found in the Brazil or the pecan nut. The forms illustrated on the plate at Fig. 4 were found in the Spanish chestnut, and exhibit the minuteness of the spores, and also show their gradual development as well as the commencement of the growth of mycelium. One body, centrally situated, shows the development of spores by gemmation. Other forms show the development of spores by septation. The spores in two cases are seen escaping from the sporidia.

M. C. Cook, in his account of the British fungi, says that the cryptogamous, unlike the phanerogamous plants, give off carbonic acid and absorb oxygen. We generally recognize such to be the case with some of the ferments, while others give off acetic acid, as was the case with the fungus found in the pecan nut, and as also is the case with the "mother" which grows upon the surface of cider when exposed to the air. There are facts which seem to show *that the only limit to the acid products of these bodies — the fnngi — is the constitution and condition of the substance in which they find lodgment and are capable of developing.* The vast number of these bodies may be inferred when it is known that mycologists at the present time, with a liberal allowance for hair-splitting, reckon among British species of flowering plants only one-fourth of the number of the fungi alone, not to mention ferns, mosses, algæ, and lichens. There are strong reasons for believing that carbonic oxide is also developed by some of these low forms of vegetable life. Would it not be proper to call them vegetable deaths? It is not clear to my mind that any of these, if left, would multiply for any length of time at the expense of the tooth, but it is perfectly clear that many of them have been introduced with particles of food, where by their presence they have exerted an injurious effect upon the tooth substance, in that they have acted to decompose the food with which they were associated, if not also to decompose portions of the tooth.

It is well known that in all fermentable substances, fermentation is started by the presence of an azotized body; that the fermentable substance changes and has formed within it, as a result of this fermentation, some acid; that fermentative changes take place with varying rapidity, according to the more or less favorable conditions of temperature and moisture; that the buccal cavity is, in a normal condition, most highly favorable to such changes; that all foods are more or less infested by some form of fungus; that the air is, to a great extent, at the sea-level, and over most of the earth's surface, so impregnated with cryptogamous spores (and all cryptogamous spores are nitrogenous) that foods can only be kept from fermentation for any length of time by destroying the azotized bodies with which they are in contact by the application of a temperature sufficiently high to destroy all such life, or by combining with them certain chemical agents. It is also well known that all foods not so protected against contact with azotized bodies will, in the presence of sufficient warmth and moisture, be liable to destructive fermentation. Hence it may be seen that wherever there are imperfections in teeth, or places where a lodgment of food or fermentable matter may occur, there will be a liability to the formation of acids, and of course to the solution of lime-salts in the contiguous portion of the tooth. So that all imperfections that cannot be so filled as to perfectly secure them from further lodgments should be so shaped as to readily be kept clean by friction. Teeth with soft enamel will naturally be the most difficult to preserve by filling, as in such teeth the crystals of enamel are more loosely put together, the spaces between them more easily penetrated by foreign substances; and a greater difficulty will be found in coapting the filling, of whatever nature, to the surfaces of such teeth than to the surfaces of the more dense and perfect. And it will be understood that the softer and more pliable the filling material, the more readily will it pass into the inequalities of the surface, and hence more perfectly protect it. But it happens, most unfortunately, that of the substances used for such filling purposes, the one most stable is the most difficult to adapt perfectly to the minute inequalities of the walls of a cavity.

With these facts in view, it is not necessary to consider gold as "incompatible with tooth-substance," in order to account for the failures which in certain cases have been attributed to it. But this difficulty attending the adaptation of gold to the walls of the cavity is easily overcome, for nature has not left us without a fitting substance to fill these minute scratches and imperfections of soft teeth, to the entire exclusion of the minute spores we have had under discussion. This substance will no more sustain the life of a fungoid spore than will carbonic monoxyde the life of a human being. It is pliant, inodorous, tasteless, non-oxydizable, non-volatile, non-irritant even to the pulps of teeth, by which it is readily absorbed, inducing in them the highest functional activity compatible with comfort to the individual. The substance is vaseline.* With the aid of vaseline the dentist will now be enabled to save the soft teeth as readily with gold as he formerly did those of denser structure.

My method of using this material is as follows:

After having prepared the cavity for filling by the removal of débris and carious walls, and by properly shaping it, I destroy the lower forms of life in the walls of the cavity, so far as I am able, by the use of a saturated aqueous solution of carbolic acid and a saturated aqueous solution of bisulphite of soda, singly or combined. I then dry the cavity with bibulous paper, and with a small wad of the same rub into its surface the vaseline, leaving a

---

* Vaseline is one of the products obtained from petroleum by repeated filtration through boneblack, according to a process patented by Robert A. Cheesebrough, and furnished by him to the profession, without a royalty.

super-abundance in the cavity until I am ready to fill. I then wipe the cavity clean, just as the steel and copper-plate printers do the face of the plate when about to print from it. And just as the ink is retained in the lines and scratches of his plate, so will the vaseline be in the porous enamel and scratches in the dentine, to the exclusion of other foreign matter.

---

ALTERATIONS OF THE SYMPATHETIC IN A CASE OF PERNICIOUS ANÆMIA. Brigidi. (*Lo Sperimentale*, *V*, '78.)

Since Birmer's discovery of certain changes in the tissues the attention of other pathologists has been directed to the origin of this anæmia. A domestic aged 53, died of a slowly developed, progressive, pernicious anæmia; post mortem section revealed an enormous deposit of fat in the panniculus adiposus, but no further changes in the heart or other viscera.

But the cardiac plexus in the fresh state showed an enormous granular proliferation, so that at many points the nerve cells were compressed; at others they were pigmented; the blood vessels were empty. In the ganglion hardened by alcohol, nerve cells were found scatteringly, their places being occupied by groups of small elements which appeared like nucleoli. These appearances led the writer to explain the pathological processes thus: The endothelium lining the capsule of the ganglion gradually enlarges, compressing the nerve cells and forming more and more granulations, a few of which take on a bronzed color whilst others undergo a fatty degeneration. At last both nerve cells and fibers succumb. The empty blood vessels of the ganglion also present an unusual proliferation of endothelium. In the immediate vicinity of the ganglion a large quantity of connective tissue, poorly supplied with nerves, is found. Inasmuch as this ganglion influences the blood supply of the chylopoëtic apparatus, digestion and assimilation are poorly performed, respiration diminishes in frequency, oxidation of blood is retarded, the fatty elements of the blood increase, fat is deposited, and the processes run in a vicious circle. (*Cin. Lancet and Clinic*, *Jan.* '79.)

## Figures and Tables

**Fig. 1 Fig. 2 Fig. 3 Fig. 4 f1:**